# Spouse support and stress: gender differences in neural measures of performance monitoring under observation of a spouse

**DOI:** 10.1093/scan/nsaf053

**Published:** 2025-05-19

**Authors:** Peter E Clayson, Kipras Varkala, Scott A Baldwin, Patrick R Steffen, Jonathan G Sandberg, Michael J Larson

**Affiliations:** Department of Psychology, University of South Florida, Tampa, FL 33620, United States; Department of Psychology, University of South Florida, Tampa, FL 33620, United States; Department of Psychology, Brigham Young University, Provo, UT 84602, United States; Department of Psychology, Brigham Young University, Provo, UT 84602, United States; School of Family Life, Brigham Young University, Provo, UT 84602, United States; Department of Psychology, Brigham Young University, Provo, UT 84602, United States; Neuroscience Center, Brigham Young University, Provo, UT 84602, United States

**Keywords:** performance monitoring, spousal support, error-related negativity (ERN), event-related potentials (ERPs), gender differences, error positivity (Pe)

## Abstract

Spousal support can mitigate stress’s impact on daily functioning and neural responses to stressors. However, the effectiveness of spousal support in reducing stress may be moderated by gender. The present study investigated the impact of observer presence in 66 heterosexual married couples, specifically a spouse or a confederate, on two neural indices of performance monitoring: early error detection [error-related negativity (ERN)] and later error awareness [error positivity (Pe)]. Contrary to predictions, ERN was consistently smaller in observed conditions, suggesting that being observed, irrespective of the observer’s identity, diminished attention to errors. Notably, only women exhibited an enhanced ERN in the presence of their spouse, suggesting gender-specific differences in neural responses to spousal support during performance monitoring. Pe was larger when completing the task in the presence of a spouse and men displayed larger Pe than women. The present findings underscore the complex role of social context in performance monitoring, challenging existing assumptions about the uniformity of neural indices of performance monitoring during observation. Findings emphasize the need to dissect the nuanced interplay between observer presence, gender differences, and performance monitoring and offer valuable insights into the social modulation of error processing, particularly in a stressful observation context.

## Introduction

Being in a securely attached romantic relationship may help mitigate the effects of stress on daily functioning. For example, the presence of a spouse mitigates neural responses to stressors, and marital satisfaction predicts the reductions in neural activity following threats (Coan et al. 2006). Alternatively, unsupportive spousal behaviours are associated with larger neural responses following participant errors (Palmwood and Simons 2021). Gender differences might moderate the relationship between spousal support and stress responses (Christakou et al. 2009, Crowley et al. 2009, Lighthall et al. 2012). Gender differences could clarify the nuanced relationship between spousal support and performance in the context of stressors, supporting the development of targeted interventions that could lead to better physical and emotional health (Holt-Lunstad et al. 2008). Therefore, we sought to determine whether gender differences moderate the impact of spousal support on neural responses related to errors when participants are under evaluation of their spouse or other-gender confederate.

The relationship between marital satisfaction and physical health is supported by psychophysiological data, which emphasize the importance of interpersonal bonds and support the secure base theory (Ainsworth et al. 2015). Satisfaction and support predict better physical health such that couples with high marital quality have lower ambulatory blood pressure than couples with low marital quality (Holt-Lunstad et al. 2008). An fMRI study assessing the impact of spousal support, via handholding during electric shock administration, observed reduced threat-related brain activity (Coan et al. 2006, see also Johnson et al. 2013). This research points to the positive impact of spousal support on stress and overall health, being dependent on the level of satisfaction obtained from a relationship. That is, the more satisfied in their relationship with their spouse, the more likely they are to have a healthier response to stressful situations.

An example of a potentially stressful experience is the evaluation of performance. Specific research on the effects of an unfamiliar observer on cognitive functioning suggests higher levels of performance anxiety and poorer performance on tests of attention, memory, and executive functions than performance under no observation (Horwitz and McCaffrey 2008). Such detrimental effects on cognitive performance are present even when the observer is a parent, sibling, close friend, spouse, or partner of the individual taking the tests (Kehrer et al. 2000). Therefore, the presence of an observer may be treated as an inherent evaluator of performance, increasing subjective feelings of stress.

An index of performance monitoring that appears sensitive to others’ evaluation is the error-related negativity (ERN), an event-related brain potential (ERP) that is larger (i.e., more negative) for error response than correct responses (Falkenstein et al. 1991, Gehring et al. 1993, 2012, Larson et al. 2014). Although the precise functional significance of ERN remains unclear (Clayson, Kappenman et al. 2021), ERN appears to represent the activity of a performance-monitoring system that serves to adjust goal-directed behaviour (Weinberg et al. 2015), which is consistent with relationships between ERN and various cognitive and motivational processes (Olvet and Hajcak 2008, Larson et al. 2012, Proudfit et al. 2013, Larson et al. 2014, Weinberg et al. 2015). Therefore, the magnitude of the ERN can be a useful index of attention to errors (Larson and Clayson 2011, Larson et al. 2012) and could be sensitive to the impact of perceived spousal support on performance monitoring.

Considering that the presence of strangers modifies neural responses to stress in participants (Coan et al. 2006), the relationship between ERN and stress during observational paradigms is worth dissecting. Findings on the relationship between ERN and stress are mixed, with increased acute stress levels with reduced (i.e. more positive) ERN amplitude in men (Hu et al. 2019), similar ERN amplitude between acute social stress and control conditions (Rodeback et al. 2020), and larger ERN in individuals with high levels of lifetime stress exposure, particularly social-evaluative threat (Banica et al. 2022). ERN tends to be larger when participants are observed by a confederate than when completing a task alone (Hajcak et al. 2005), although the influence of the observer’s identity was not examined. In a study in which participants were observed by a romantic partner, ERN amplitude was similar under observation and when alone (Palmwood and Simons 2021), suggesting that the identity of the observer influences whether changes in ERN are observed under observation. However, Palmwood and Simons (2021) did not compare partner observation to observation by unfamiliar people (i.e. confederates), preventing an examination of whether the observer’s identity moderates the influence of observer evaluation on performance monitoring. It is possible that heightened feelings of stress due to the presence of a confederate contribute to overactive error monitoring under evaluation, but only when the observer is a stranger.

The error positivity (Pe) is another index of performance monitoring that might be sensitive to performance evaluation. Pe is a slow tonic waveform that peaks around 300 ms and is more positive for error responses than correct responses (Falkenstein et al. 1991, O'Connell et al. 2007). Although ERN indexes early error detection, Pe reflects later conscious processing of errors and is relatively independent of ERN (Falkenstein et al. 2000, Shalgi et al. 2009). ERN in participants differentiated between harmful and nonharmful consequences of errors inflicted upon an observer, while Pe amplitude was similar across conditions (de Bruijn et al. 2020). Pe amplitude is also reduced following acute social stress manipulations, whereas ERN amplitude is not significantly affected (Rodeback et al. 2020). Taken together, error significance appears to affect early error detection, whereas stress influences later conscious processing. Therefore, ERN and Pe might show distinct relationships with social context, reflecting different stages of performance monitoring that are differentially sensitive to social and affective factors.

Gender differences are contended in research trying to understand the interaction between spousal support and cognition (Christakou et al. 2009, Crowley et al. 2009, Lighthall et al. 2012). Husbands reported that they perceive their wives as providing more quality support than they provide (Cutrona 1996); however, a diary study observed quality of support provided did not actually differ between genders (Neff and Karney 2005). The diary study indicated that the timing of support was different for husbands and wives. When a partner was experiencing stress, wives provided only positive support and withheld negative criticism, whereas husbands provided positive support and criticisms (Neff and Karney 2005). It may be a matter of the mere perception of support, and the degree to which support was felt had a greater association with well-being and marital satisfaction for wives as opposed to husbands (Acitelli and Antonucci 1994).

In relation to ERN and Pe, gender differences remain unclear, with some studies showing that men, compared to women, exhibit a larger ERN (Larson et al. 2011, Fischer et al. 2016, 2017), smaller ERN (Themanson et al. 2011), or similar ERN (Weinberg et al. 2010, Moran et al. 2012). Results are similarly mixed for Pe, with some studies showing that men, compared to women, show larger Pe amplitude (Larson et al. 2011) or no differences in amplitude (Themanson et al. 2011, Fischer et al. 2016), although studies to date on Pe are sparse. Study-to-study differences may explain the conflicting findings. Meta-analytic research indicates that gender moderates the ERN-anxiety relationship, such that the relationship is stronger in women than in men with larger ERN related to higher anxiety symptoms (Moser et al. 2016). Considering gender differences in ERN and in the impact of spousal support on cognition, gender could affect the influence of spousal support on performance monitoring as indexed by ERN.

The primary aim of the present study was to determine whether spousal support mitigates the impact of stress during performance monitoring under observation. Participants completed a flanker task alone, while being observed by an other-gender confederate, and while being observed by their other-gender spouse. We predicted that participants would show larger ERN when observed than when completing a task alone. We also predicted that ERN would be smaller when completing the task in the presence of a spouse than in the presence of an unfamiliar confederate, and we examined whether gender moderated this relationship. Because research on Pe and stress is sparse, we did not have specific predictions for Pe, and analyses are considered exploratory.

## Materials and methods

### Participants

A total of 132 people from 66 heterosexual married couples participated in the study. Inclusion criteria were 18-55 years old, right-handed, and native English speakers. Exclusion criteria were a psychiatric diagnosis, self-reported alcohol or substance abuse within the past year, history of learning disability, Attention-Deficit/Hyperactivity Disorder (ADHD), neurological disorder (e.g. traumatic brain injury, seizure disorder, stroke), or current use of antiepileptic medication. Participants were recruited through fliers in the local community and provided written consent for all study procedures. Seven participants were excluded due to not having any EEG data that survived artefact rejection (see Electrophysiological Recording and Data Reduction section). Therefore, final study enrolment included a total of 125 participants (65 women, 60 men) from 66 couples—some couples had partial data due to the exclusion of seven participants. All participants were in their first marriage and were relatively recently married (*M *= 2.2 years^1^, *SD *= 4.0 years).

### Procedures

Each person from each couple participated in a single experimental session consisting of two main parts: (1) recording of event-related potentials (ERPs) and response times during the completion of a flanker task and (2) completion of self-report questionnaires. The computerized task was administered three times: (1) baseline (no observer), (2) confederate observer (a member of the research team matched with spouse sex), and (3) spouse observer. The baseline condition was always completed first. The order of spouse completion (husband vs. wife) and the order of the observer completion (confederate vs. spouse) were counterbalanced across couples. The spouse which was not participating in the computerized task was taken to a separate room and finished the self-report questionnaire portion of the study. Each session required approximately three hours of participation per couple and couples were provided $50 monetary compensation for their time.

### Self-report measures

Self-report measures were included in the study to evaluate the effects of personal (e.g. mood) and relationship factors (e.g. marital satisfaction) on study outcomes. Most relevant to the current manuscript were the Interpersonal Reactivity Index (IRI; Davis 1980), used to assess empathy and includes four subscales: Perspective Taking (PT), Fantasy (FS), Empathic Concern (EC), and Personal Distress (PD), and the Revised Dyadic Adjustment Scale (RDAS; Busby et al. 1995), used to assess marital adjustment and satisfaction. Descriptive statistics are provided in Table 1, and analyses with self-report measures used are provided in the supplementary material.

**Table 1.** Summary information for demographics, self-report, behavioural and ERP data.

	Women		Men	
	*M*	*SD*	*M*	*SD*
Age (years)	24	6	25	5
Personal education (years)^a^	16	1	16	2
BDI-II	6	5	5	4
STAI: State	32	9	30	7
STAI: Trait	36	8	34	7
PANAS: Negative affect	18	5	18	4
PANAS: Positive affect	34	6	35	6
IRI: Perspective taking	18	3	17	3
IRI: Fantasy	25	6	23	6
IRI: Empathic concern	29	3	26	5
IRI: Personal distress	18	5	15	4
RDAS	54	5	54	6

	Alone		Spouse		Confederate		Alone		Spouse		Confederate	
	*M*	*SD*	*M*	*SD*	*M*	*SD*	*M*	*SD*	*M*	*SD*	*M*	*SD*
Behavioural data												
Congruent RT (ms)	391	27	377	26	380	29	374	31	364	28	365	29
Incongruent RT (ms)	458	35	434	31	437	32	435	34	417	35	421	35
Congruent accuracy	0.97	0.02	0.98	0.01	0.98	0.02	0.98	0.02	0.98	0.02	0.98	0.02
Incongruent accuracy	0.90	0.07	0.92	0.06	0.92	0.05	0.92	0.05	0.92	0.07	0.91	0.07
ERP												
Correct trials	735	41	739	34	754	103	747	34	750	35	739	50
Error trials	47	37	36	25	39	27	39	23	43	32	42	32
CRN (µV)	2.6	1.8	3.9	1.9	4.0	1.0	2.9	2.2	4.3	2.3	4.2	2.4
ERN (µV)	−0.3	2.5	0.2	2.8	0.8	2.6	−0.5	2.6	0.5	2.8	0.1	3.0
Correct-Trial Pe (µV)	−0.7	1.1	−0.4	1.2	−0.3	1.3	0.4	1.3	1.0	1.3	1.0	1.3
Error-Trial Pe (µV)	3.0	2.9	0.9	1.3	3.5	2.5	4.7	2.5	5.8	2.8	5.6	3.2
	*N*		*n*		*n*		*n*		*N*		*n*	
Number of participants retained for analysis	64		58		61		57		59		59	

*Note*: BDI-II = Beck Depression Inventory, 2^nd^ Edition; STAI = State-Trait Anxiety Inventory; PANAS = Positive and Negative Affect Schedule; IRI = Interpersonal Reactivity Index; RDAS = Revised Dyadic Adjustment Scale; CRN = correct-related negativity; ERN = error-related negativity; Pe = error positivity. a Data are missing for three women and five men.

**Table 2.** Estimates from location-scale multilevel model predicting error-related negativity amplitude.

Predictor	Estimate	*SE*	95% CrI
Location portion			
Intercept	2.58	0.24	2.11, 3.06
Event (error)	−2.90	0.27	−3.43, −2.37
Confederate	1.30	0.03	1.23, 1.36
Spouse	1.23	0.03	1.17, 1.30
Gender (man)	0.46	0.33	−0.19, 1.10
Event × Confederate	−0.31	0.14	−0.58, −0.03
Event × Spouse	−0.63	0.15	−0.92, −0.35
Event × Gender	−0.53	0.39	−1.31, 0.25
Gender × Confederate	−0.12	0.05	−0.21, −0.03
Gender × Spouse	0.03	0.05	−0.06, 0.12
Event × Confederate × Gender	−0.32	0.20	−0.71, 0.07
Event × Spouse × Gender	0.28	0.21	−0.13, 0.68
Scale portion (*SD*)			
Intercept	1.53	0.02	1.49, 1.57
Event	−0.02	0.02	−0.05, 0.02
Confederate	0.08	0.00	0.07, 0.09
Spouse	0.07	0.00	0.06, 0.08
Gender	0.03	0.03	−0.02, 0.09
Event × Confederate	0.03	0.02	−0.01, 0.07
Event × Spouse	0.03	0.02	−0.01, 0.07
Event × Gender	0.03	0.02	−0.02, 0.07
Gender × Confederate	−0.08	0.01	−0.09, −0.06
Gender × Spouse	−0.06	0.01	−0.07, −0.04
Event × Confederate × Gender	−0.02	0.03	−0.08, 0.04
Event × Spouse × Gender	−0.03	0.03	−0.09, 0.03
Random effects			
Participants			
Mean structure			
Intercept	1.83	0.17	1.51, 2.17
Event	2.05	0.16	1.76, 2.38
Variance structure (*SD*)			
Intercept	0.15	0.01	0.13, 0.17
Event	0.05	0.01	0.02, 0.07
Couples			
Mean structure			
Intercept	0.70	0.32	0.06, 1.31
Event	0.40	0.27	0.02, 0.98
Variance structure (*SD*)			
Intercept	0.03	0.02	<0.01, 0.07
Event	0.03	0.01	<0.01, 0.05

*Note:* Parameters in standard deviations (*SD*) units are shown on a log scale. See Supplementary Table 9 for covariance and correlations among random intercepts and slopes across location and scale portions of the model. *SE* = standard error; 95% CrI = 95% credible interval.

### Experimental tasks

Participants completed a modified version of the flanker task; the task is described briefly here and in full in the supplementary material. Each trial comprised a congruent or an incongruent stimulus. Participants were instructed to respond as quickly and accurately as possible. Both confederate and spouse observation conditions included the observer seated just behind and to the left of the participant. Observers were given headphones that made an audible ‘ding’ each time the participant made an error. Only the observer heard the ‘ding’ through the headphones.

### Electrophysiological data recording and reduction

EEG data recording and reduction is described in detail in the supplementary material and briefly outlined below. EEG was using a 129-channel hydrocel geodesic sensor net and Electrical Geodesics, Inc. (EGI; Eugene, OR) amplifier system. Continuous EEG was filtered offline with half-amplitude cut-offs at 0.01 and 15 Hz. Epochs were extracted from 400 ms prior to the participant’s button press to 800 ms following the button press. Ocular artefact was removed using independent components analysis, and bad channels were identified and interpolated. EEG was rereferenced to an average reference, and the period from 400 ms to 200 ms prior to participant response was used for baseline adjustment. ERN was extracted as the average activity over fronto-medial sites [6 (FCz), 129 (Cz), 7, 106; for electrode configuration, see Clayson and Larson 2013] and quantified using a time-window mean amplitude (average activity from 0 to 100 ms). Pe was extracted as the average activity over centro-parietal sites [54, 55, 61, 62 (Pz), 78, 79] and quantified using a time-window mean amplitude (average activity from 200 to 400 ms).

### Data analysis

#### Psychometric internal consistency

Estimates of psychometric internal consistency are included (Clayson and Miller 2017a, 2017b, Clayson 2024b, Heindorf et al. 2025) and are described in the supplementary material.

#### Multilevel models

Multilevel models are well-suited for single-trial ERP scores (Brush et al. 2018, Volpert-Esmond et al. 2018, Clayson and Larson 2019, Volpert-Esmond et al. 2021, Park et al. 2024, Clayson, Rocha et al. 2024, Clayson, Shuford et al. 2024, Clayson, Baldwin et al. 2025, Clayson, McDonald et al. 2025, Holbrook et al. 2025) and data nested within couples (Bartle-Haring et al. 2020). Single-trial scores were nested within participants nested within married couples and multilevel models account for the nested nature of the data. These models also account for the unbalanced nature of ERP data due to trials by partially pooling information across parameters to improve their estimation (Gelman and Hill 2006, Gelman et al. 2012). This is beneficial here, where some participants have few trials in certain sessions (alone vs. with spouse vs. with confederate).

Location-scale multilevel models^2^ were used to simultaneously estimate mean and residual variances (Walters et al. 2018), and these models have been successfully applied in studies of ERPs (Clayson, Brush et al. 2021, 2022b, 2024b, Holbrook et al. 2025; for commentary on the use of these models in studies of ERPs; see Volpert-Esmond 2022). These models expand the multilevel structure into the scale (i.e. variance) part of the model, which allows for the simultaneous modelling of means (i.e. location) and within-person variances (i.e. scale). The multilevel location-scale model allows a multilevel structure for the residual variance, including both fixed and random effects.

Multilevel location-scale models used the same predictors on the location and scale portions. The base model for ERP data included a population intercept, a fixed effect for event [correct (reference), error], random intercepts for participants and for couples, random slopes for event within participants and within couples, and covariances between random intercepts and slopes. In the base model for reaction time (RTs), there was a fixed effect for congruency [congruent (reference), incongruent] instead of a fixed effect for event. Three separate models were run that additionally included (1) a fixed effect for session [alone (reference), with confederate, with spouse], (2) a fixed effect for gender [woman (reference), man], or (3) fixed effects for session and gender and their interaction. The priors used for model fitting are described in the supplementary material.

Models were fit in R Development Core Team (2021) using the package *brms* (Bürkner, 2017, 2018), which is a front-end wrapper for Stan Development Team (2021).

Leave-one-out cross-validation via Pareto smoothed importance sampling (PSIS-LOO-CV) was used to compare model fit (Vehtari et al. 2017) using the *loo* package in *R* (Vehtari et al. 2022). All post-estimation contrasts were performed on the posterior estimates from the multilevel models. Effects were interpreted when the post-estimation contrasts excluded zero from the 95% credible intervals. Scale effects are reported in standard deviations on the log scale.

#### Exploratory analyses

Scores from self-report measures were included as covariates in the best-fitting models for each dependent variable. Scores from self-report measures were first *z*-score transformed to a mean of zero and a standard deviation of one prior to analysis in multilevel models. The covariates were included in the highest-order interaction and all lower-order interactions for each model. Model fits were subsequently examined against the best fitting models without covariates using PSIS-LOO-CV. Exploratory analyses are described in the supplementary material.

## Results

**Figure 1.**
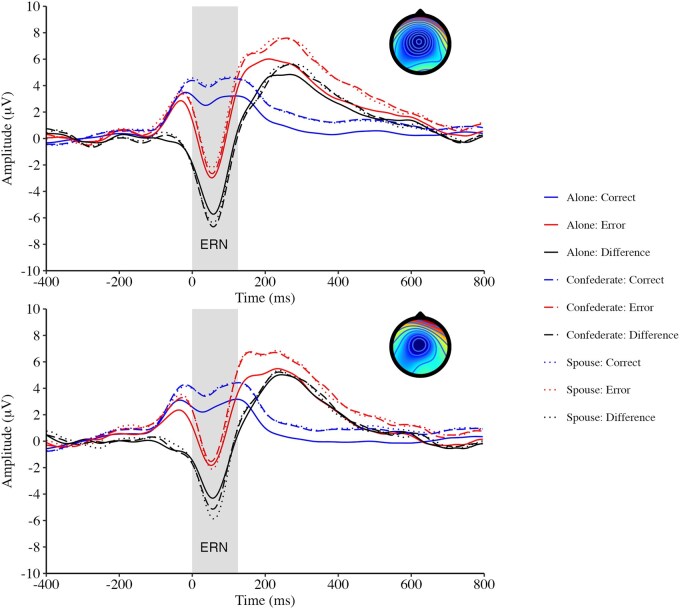
Grand average waveforms for the ERN separately for men (top row) and women (bottom row). Waveforms are shown for fronto-central activity across four sites for ERN on the top row, and activity corresponding to ERN is shown in gray. Topographical maps showing the frontocentral scalp distribution of ERN for each group are shown in the top-right corner of each plot, and the maps represent the average ERN difference activity (error minus correct) from 0 to 100 ms for waveforms during the alone condition.

**Figure 2.**
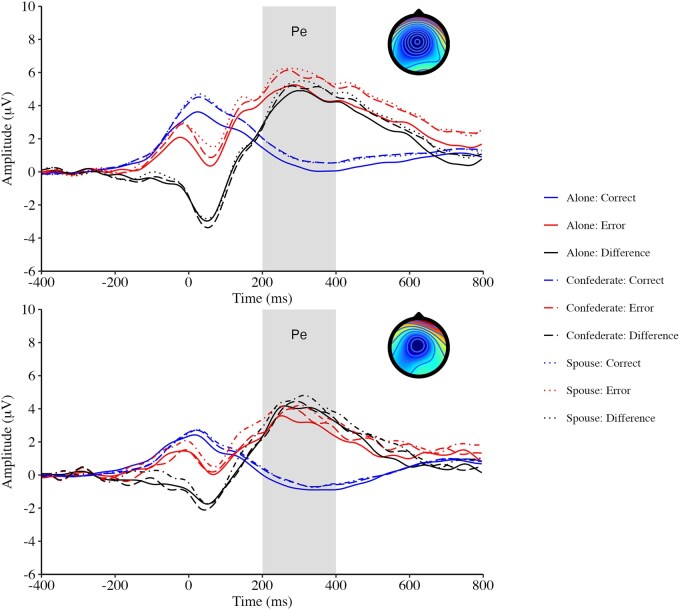
Grand average waveforms for the Pe separately for men (top row) and women (bottom row). Waveforms are shown for centro-parietal activity across six sites for Pe, and activity corresponding to Pe is shown in gray. Topographical maps showing the frontocentral scalp distribution of Pe for each group are shown in the top-right corner of each plot, and the maps represent the average Pe difference activity (error minus correct) from 200 to 400 ms for waveforms during the alone condition.

Summary demographic, self-report, behavioural, and ERP data are reported in Table 1. Grand average ERP waveforms are shown in Figs. 1 and 2.

### Model selection

We first determined the best fitting models for RTs, ERN, and Pe, and the summary statistics for these model comparisons are reported in Supplementary Table 1. The best fitting model for RTs, ERN, and Pe was a model including the three-way interaction for Congruency/Event × Session × Gender; differences in expected log predictive density (${\hat{\Delta \mathrm{elpd}}}_{\mathrm{loo}}$) were greater than 64 for RTs, 78 for ERN, and 21 for Pe in favour of the three-way interaction models (see Supplementary Table 1). These findings indicate that the inclusion of the three-way interaction and each lower-level interaction and main effect showed the highest predictive accuracy and generalizability to other unseen data. Therefore, the model including the three-way interaction was interpreted for each dependent variable. Findings for RTs are described in the supplementary material, as the focus of the present manuscript is on ERN on Pe.

### Error-Related negativity

**Figure 3.**
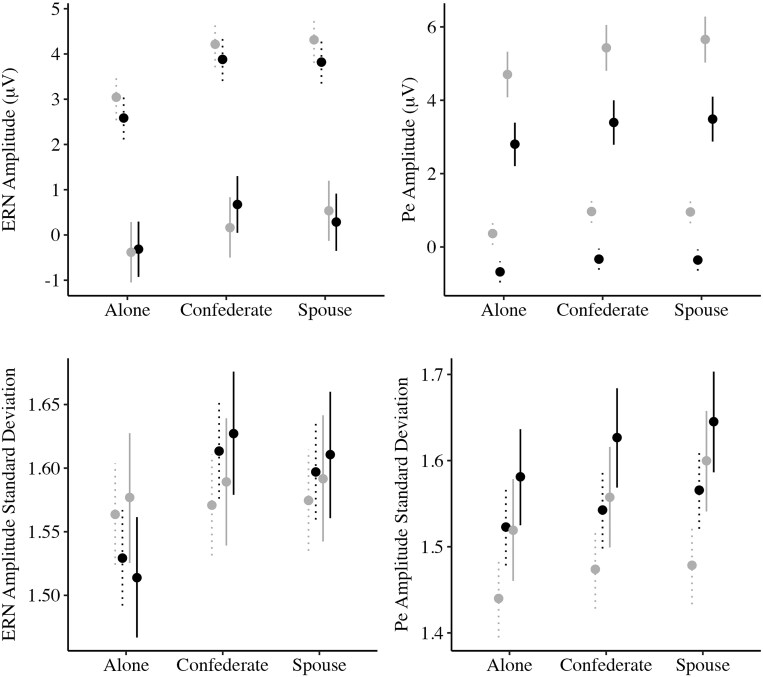
Point estimates for posterior samples with their 95% credible intervals. Estimates of average error-related negativity (ERN; top left column) and error positivity (Pe; top right column) are shown on the top, and estimates of the ERN standard deviations (bottom left) and Pe standard deviations (bottom right) on the log scale are shown on the bottom.

Parameter estimates from the location-scale model predicting ERN are shown in Table 2 and Fig. 3. The unit of measurement for the 95% credible intervals for ERN and Pe is µV for the location portion of the model. Post-estimation contrasts for the scale portions of the model are reported in terms of standard deviations on the log scale.

#### Location

Error trials were larger (i.e. more negative) than correct trials (95% CrI: −3.87, −3.09). Overall, ERN was smaller (i.e. less negative) when being watched by a confederate (95% CrI: 0.90, 1.10) or a spouse (95% CrI: 0.90, 1.11) than when completing the flanker task alone. ERN was similar in amplitude when being watched by a confederate and by a spouse (95% CrI: −0.11, 0.10). Women and men showed similar overall ERN amplitude (95% CrI: −0.50, 0.81).

There were Event × Confederate (confederate vs. alone) and Event × Spouse (spouse vs. alone) interactions, such that ΔERN (error minus correct) was larger when being observed by a confederate or a spouse than when completing the task alone (95% CrI: −0.67, −0.27; 95% CrI: −0.69, −0.29, respectively). ΔERN was similar during the confederate and spouse conditions (95% CrI: −0.18, 0.23). The Event × Gender interaction did not indicate gender differences in ΔERN (95% CrI: −1.29, 0.21). Gender differences in ΔERN were not observed when completing the task alone (95% CrI: −1.31, 0.25), with a spouse (95% CrI: −1.03, 0.54), or with a confederate (95% CrI: −1.63, −0.05).

The three-way interactions did not yield differences for the confederate vs. alone (95% CrI: −0.71, 0.07) or spouse vs. alone (95% CrI: −0.13, 0.68) contrasts, but there were differences observed for the confederate vs. spouse contrast (95% CrI: −1.00, −0.19). Women showed differences in the Event × Confederate/Spouse contrast (95% CrI: 0.25, 0.63), but there was no such effect for men. ΔERN was larger for women during the spouse condition than during the confederate condition.

#### Scale

Correct and error trials showed similar variability in ERN amplitudes (95% CrI: −0.01, 0.02). Variability was larger when completing the task with a confederate (95% CrI: 0.04, 0.07) or a spouse (95% CrI: 0.03, 0.06) than when completing the flanker task alone. Variability was similar across confederate and spouse conditions (95% CrI: −0.01, 0.02). Gender differences were not observed for variability in ERN scores (95% CrI: −0.06, 0.05).

There were Event × Confederate (confederate vs. alone) and Event × Spouse (spouse vs. alone) interactions. There was less variability in ΔERN during the alone condition than during the confederate condition (95% CrI: 0.03, 0.09) or the spouse condition (95% CrI: 0.03, 0.09). ΔERN variability was similar across the confederate and spouse conditions (95% CrI: −0.02, 0.04). The Event × Gender interaction did not indicate gender differences in ΔERN variability (95% CrI: −0.02, 0.04). Gender differences in overall ERN variability were not observed when completing the task alone (95% CrI: −0.01, 0.11), with a spouse (95% CrI: −0.10, 0.02), or with a confederate (95% CrI: −0.08, 0.04).

The three-way interactions did not yield differences for the confederate vs. alone (95% CrI: −0.08, 0.04), spouse vs. alone (95% CrI: −0.09, 0.03), or confederate vs. spouse contrasts (95% CrI: −0.06, 0.06).

#### Interim summary

Overall, ERN was smaller and ΔERN was larger when being observed than when completing the task alone, and there were no gender differences in overall ERN or ΔERN. Only women showed larger ΔERN during the spouse condition than during the confederate condition.

ERN score variability was similar across correct and error trials, and there was greater overall variability in ERN and less ΔERN variability when the participant was observed than when the participant was alone. Gender differences were not observed for the scale portion of the model.

### Error positivity

Parameter estimates from the location-scale model predicting Pe are shown in Table 3 and Fig. 3.

**Table 3.** Estimates from location-scale multilevel model predicting error positivity amplitude.

Predictor	Estimate	*SE*	95% CrI
Location portion			
Intercept	−0.68	0.15	−0.97, −0.39
Event (error)	3.48	0.29	2.91, 4.05
Confederate	0.35	0.03	0.29, 0.40
Spouse	0.32	0.03	0.26, 0.38
Gender (man)	1.04	0.21	0.64, 1.44
Event × Confederate	0.25	0.14	−0.03, 0.53
Event × Spouse	0.36	0.15	0.07, 0.66
Event × Gender	0.86	0.42	0.03, 1.66
Gender × Confederate	0.26	0.04	0.17, 0.34
Gender × Spouse	0.27	0.04	0.19, 0.35
Event × Confederate × Gender	−0.12	0.20	−0.51, 0.27
Event × Spouse × Gender	0.00	0.20	−0.40, 0.40
Scale portion (*SD*)			
Intercept	1.52	0.02	1.48, 1.57
Event	0.06	0.02	0.02, 0.09
Confederate	0.02	0.00	0.01, 0.03
Spouse	0.04	0.00	0.03, 0.05
Gender	−0.08	0.03	−0.14, −0.02
Event × Confederate	0.03	0.02	−0.01, 0.07
Event × Spouse	0.02	0.02	−0.02, 0.06
Event × Gender	0.02	0.03	−0.03, 0.07
Gender × Confederate	0.01	0.01	0.00, 0.03
Gender × Spouse	0.00	0.01	−0.02, 0.01
Event × Confederate × Gender	−0.02	0.03	−0.08, 0.04
Event × Spouse × Gender	0.02	0.03	−0.04, 0.08
Random effects			
Participants			
Mean structure			
Intercept	1.14	0.08	0.98, 1.31
Event	2.20	0.17	1.86, 2.55
Variance structure (*SD*)			
Intercept	0.17	0.01	0.14, 0.19
Event	0.08	0.01	0.06, 0.10
Couples			
Mean structure			
Intercept	0.30	0.17	0.02, 0.65
Event	0.48	0.30	0.03,1.11
Variance structure (*SD*)			
Intercept	0.04	0.03	<0.01, 0.09
Event	0.01	0.01	<0.01, 0.04

*Note:* Parameters in standard deviations (*SD*) units are shown on a log scale. See Supplementary Table 9 for covariance and correlations among random intercepts and slopes across location and scale portions of the model. *SE* = standard error; 95% CrI = 95% credible interval.

#### Location

Error-trial Pe was larger (i.e. more positive) than correct-trial Pe (95% CrI: 3.68, 4.51). Pe was larger when being watched by a confederate (95% CrI: 0.47, 0.66) or a spouse (95% CrI: 0.54, 0.74) than when completing the task alone, but Pe was similar in amplitude when being watched by a confederate and by a spouse (95% CrI: −0.17, 0.03). Men showed larger overall Pe than women (95% CrI: 1.13, 2.12).

There was an Event × Spouse (spouse vs. alone) interaction, such that ΔPe (error minus correct) was larger when completing the task with a spouse than when completing the task alone (95% CrI: 0.16, 0.56). Differences were not observed for the Event × Confederate (confederate vs. alone) interaction (95% CrI: −0.01, 0.38) or the Event × Confederate/Spouse interaction (95% CrI: −0.38, 0.02). ΔPe was larger for men than for women (95% CrI: 0.02, 1.60). Gender differences in ΔPe were observed when completing the task alone (95% CrI: 0.03, 1.66) and when completing the task with a spouse (95% CrI: 0.02, 1.68), such that men showed larger ΔPe than women during alone and spouse conditions. Gender differences were not observed for the confederate condition (95% CrI: −0.10, 1.56).

The three-way interactions did not yield differences for the confederate vs. alone (95% CrI: −0.51, 0.27), spouse vs. alone (95% CrI: −0.40, 0.40), or confederate vs. spouse contrasts (95% CrI: −0.53, 0.28).

#### Scale

Error trials showed more Pe variability than correct trials (95% CrI: 0.06, 0.10). Variability was larger when completing the flanker task with a confederate (95% CrI: 0.02, 0.05) or a spouse (95% CrI: 0.04, 0.07) than when completing the task alone, and variability in overall Pe scores was smaller when completing the task with a spouse than with a confederate (95% CrI: −0.04, −0.01). Women showed more variability in Pe scores than men (95% CrI: −0.13, −0.004).

The Event × Spouse (spouse vs. alone) interaction indicated greater ΔPe variability when completing the task with a spouse than completing the task alone (95% CrI: 0.001, 0.06). The Event × Confederate (confederate vs. alone) and Event × Confederate/Spouse interactions did not yield differences (95% CrI: −0.01, 0.04; 95% CrI: −0.04, 0.01, respectively). The Event × Gender interaction did not indicate gender differences ΔPe variability (95% CrI: −0.02, 0.06). Gender differences in ΔPe variability were not observed when completing the task alone (95% CrI: −0.03, 0.07), with a spouse (95% CrI: −0.01, 0.09), or with a confederate (95% CrI: −0.05, 0.05).

The three-way interactions did not yield differences for the confederate vs. alone (95% CrI: −0.08, 0.04), spouse vs. alone (95% CrI: −0.04, 0.08), or confederate vs. spouse contrasts (95% CrI: −0.10, 0.02).

#### Interim summary

Overall, Pe was larger when being observed than when completing the task alone. ΔPe was larger when completing the task with a spouse than when completing the task alone (differences were not observed for confederate vs. spouse or confederate vs. alone). Regarding gender differences, men showed larger overall Pe and ΔPe than women, gender differences in ΔPe were specific to alone and spouse conditions.

Error-trial variability was higher than correct-trial variability for Pe, and there was greater variability when completing the task with an observer than when completing the task alone. Women showed more variability in overall Pe than men. There was more variability in ΔPe when completing the task with a spouse than when completing the task alone (differences were not observed for confederate vs. spouse or confederate vs. alone).

## Discussion

The present study examined whether the presence of a spouse attenuates neural measures of performance monitoring relative to a confederate observer and whether gender moderates these relationships. Contrary to predictions, ERN, an index of early error detection, was smaller under both spousal and confederate observation than during solitary task performance, suggesting that the presence of an observer, regardless of their relationship with the participant, reduces attention to errors. Only women showed an enhanced ERN in the presence of a spouse, an effect in the opposite direction from what was predicted. Furthermore, results showed that Pe, a measure of conscious error awareness, was larger during observed conditions, regardless of the type of observer. These findings highlight the role that social context plays during performance monitoring under evaluation, offering implications for how people perceive and react to errors in the presence of others.

ERN was smaller under confederate and spousal observation than when alone, suggesting that any observer leads to reduced attention to errors. This reduction may occur because being observed introduces cognitive demands, such as concerns about social evaluation, which divert attention from error monitoring. Since high anxiety is linked to larger ERN (Hajcak et al. 2019), present findings suggest reduced neural responses to errors during social evaluation under observation by a spouse or other-gender confederate (Coan et al. 2006). Present findings contradict smaller studies, one showing larger ERN under same-gender confederate observation (*n *= 18; Hajcak et al. 2005) and another showing similar ERN amplitudes when observed by a romantic partner or when alone (*n *= 28; Palmwood and Simons 2021). Hajcak et al. used mostly male participants and male confederates, while Palmwood and Simons lacked a confederate condition, limiting their ability to examine the impact of observer’s identity. In contrast, the present study used both an other-gender confederate and an other-gender spouse, and methodological differences (e.g. sample size, gender dynamics) may account for discrepancies with the two studies.

Notably, differences in ERN between the spouse and confederate observer conditions were only observed for women; women showed larger ΔERN in the presence of a spouse than in the presence of a confederate. This suggests that women may be more sensitive to spousal observation than men, possibly leading to greater attention to mistakes as indexed by ERN. Considering that the gender groups reported similar marital adjustment and satisfaction on the RDAS, and exploratory analyses showed that RDAS scores were not related to ERN, it seems unlikely that poor spousal support contributed to observed gender differences. Present findings are inconsistent with observations that women benefit more from spousal support than men during times of stress (Acitelli and Antonucci 1994, Neff and Karney 2005), indicating that the effect of spousal observation on performance monitoring likely involve gender-specific factors, such as the gender of the participant, spouse, and confederate.

Pe findings provide further context for interpreting ERN findings. ΔPe was larger when completing the task with a spouse than when alone, suggesting that participants demonstrated increased later awareness of errors when being observed by a spouse. Since the Pe reflects later, conscious error processing and the affective evaluation of errors (Overbeek et al. 2005, Shalgi et al. 2009), this increase might be due to heightened concern about a spouse’s perception. The fact that Pe did not increase under confederate observation indicates that the identity of the observer plays a crucial role in affecting conscious error processing. Gender differences were observed with men showing a larger Pe than women, and this finding is consistent with one study (Larson et al. 2011), although this finding is inconsistent (Themanson et al. 2011, Härpfer et al. 2020, Hsieh et al. 2021). Further research on spousal support could clarify how gender moderates the relationship between spousal support and performance monitoring.

The present study had limitations. First, the ‘feeling’ of being observed was not directly measured. Several studies have used financial (Koban et al. 2010, 2012, Varkala 2023) and physically aversive (de Bruijn et al. 2020) impacts on the observer as a result of a participant’s performance to make the presence of an observer salient. Objective measures of stress were not collected, and it is plausible that some individuals might have felt positive emotions while their spouse was in the room. Second, although it is common to use a single task to record ERPs, different tasks can moderate ERP effects (Clayson, Rocha et al. 2022; Clayson 2024a), and other tasks might show stronger (or weaker) gender differences as they relate to observers; research might consider multiverse-like approaches that consider many reasonable approaches for recording and analysing the same phenomena (Clayson 2024a). Third, participants were relatively young and early in their married relationships, limiting the generalizability of the findings; future research might consider additional information about marriages, such as number of years married. Fourth, the study was not preregistered, and follow-up research might consider using a registered report format (Clayson, Keil et al. 2022). Finally, participants were excluded if they self-reported current mental illness, but lifetime psychiatric diagnoses was not assessed. Despite these limitations, the present study demonstrated several strengths, including a large sample size for an ERP study (Clayson 2019; Kissel 2023) and the first examination of how gender moderates performance-monitoring ERP components in married participants.

Taken together, the present findings provide evidence that the presence of observers, including spouses, attenuates neural responses associated with early error detection, as evidenced by reduced ERN in observed conditions, and enhanced Pe when observed by a spouse, but not a confederate. Only women showed larger ΔERN during the spouse condition than during the confederate condition, and men showed larger ΔPe during alone and spouse conditions. Present findings also highlight the need to consider gender differences to understand the impact of an observer on performance monitoring. This study expands our understanding of social influences on performance monitoring, offering insights for future research at the intersection of social psychology and neuroscience.

## Supplementary Material

nsaf053_Supplementary_Data

## Data Availability

Data underlying this article are available from the corresponding author upon reasonable request. Because participants did not consent to public release of data, access is limited to qualified researchers for purposes such as verifying the findings, in line with APA ethical guidelines.

## Footnotes

1 Data were missing for one couple.

2 A discussion of location-scale models and their application to ERPs is described in the supplementary material of Clayson et al. (2022).
