# Supplementary material for: Spouse support and stress: gender differences in neural measures of performance monitoring under observation of a spouse
Source: Soc Cogn Affect Neurosci. 2025 May 19;20(1):nsaf053. doi: 10.1093/scan/nsaf053 (PMC12342920; doi:10.1093/scan/nsaf053)
Supplement: nsaf053_Supplementary_Data [file nsaf053_supplementary_data.docx]

Spouse Support and Stress: Gender Differences in Neural Measures of Performance Monitoring Under Observation of a Spouse

Peter E. Clayson^1^, Kipras Varkala^1^, Scott A. Baldwin^2^, Patrick R. Steffen^2^, Jonathan G. Sandberg^3^, and Michael J. Larson*^2, 4^

^1^ Department of Psychology, University of South Florida, Tampa, FL

^2^ Department of Psychology, Brigham Young University, Provo, UT

^3^ School of Family Life, Brigham Young University, Provo, UT

^4^ Neuroscience Center, Brigham Young University, Provo, UT

Word Count: 4,972

**Corresponding author at:* Department of Psychology and Neuroscience Center, Brigham Young University, 245 TLRB, Provo UT 84602. Email: [michael_larson@byu.edu](mailto:michael_larson@byu.edu)

Abstract

Spousal support can mitigate stress’s impact on daily functioning and neural responses to stressors. However, the effectiveness of spousal support in reducing stress may be moderated by gender. The present study investigated the impact of observer presence in 66 heterosexual married couples, specifically a spouse or a confederate, on two neural indices of performance monitoring: early error detection (error-related negativity [ERN]) and later error awareness (error positivity [Pe]). Contrary to predictions, ERN was consistently smaller in observed conditions, suggesting that being observed, irrespective of the observer’s identity, diminished attention to errors. Notably, only women exhibited an enhanced ERN in the presence of their spouse, suggesting gender-specific differences in neural responses to spousal support during performance monitoring. Pe was larger when completing the task in the presence of a spouse and men displayed larger Pe than women. The present findings underscore the complex role of social context in performance monitoring, challenging existing assumptions about the uniformity of neural indices of performance monitoring during observation. Findings emphasize the need to dissect the nuanced interplay between observer presence, gender differences, and performance monitoring and offer valuable insights into the social modulation of error processing, particularly in a stressful observation context.

Keywords: performance monitoring; spousal support; error-related negativity (ERN); event-related potentials (ERPs); gender differences; error positivity (Pe)

Being in a securely-attached romantic relationship may help mitigate the effects of stress on daily functioning. For example, the presence of a spouse mitigates neural responses to stressors, and marital satisfaction predicts the reductions in neural activity following threats (Coan et al., 2006). Alternatively, unsupportive spousal behaviors are associated with larger neural responses following participant errors (Palmwood & Simons, 2021). Gender differences might moderate the relationship between spousal support and stress responses (Christakou et al., 2009; Crowley et al., 2009; Lighthall et al., 2012). Gender differences could clarify the nuanced relationship between spousal support and performance in the context of stressors, supporting the development of targeted interventions that could lead to better physical and emotional health (Holt-Lunstad et al., 2008). Therefore, we sought to determine whether gender differences moderate the impact of spousal support on neural responses related to errors when participants are under evaluation of their spouse or other-gender confederate.

The relationship between marital satisfaction and physical health is supported by psychophysiological data, which emphasize the importance of interpersonal bonds and support the Secure Base Theory (Ainsworth et al., 2015). Satisfaction and support predict better physical health such that couples with high marital quality have lower ambulatory blood pressure than couples with low marital quality (Holt-Lunstad et al., 2008). An fMRI study assessing the impact of spousal support, via handholding during electric shock administration, observed reduced threat-related brain activity (Coan et al., 2006; see also Johnson et al., 2013). This research points to the positive impact of spousal support on stress and overall health being dependent on the level of satisfaction obtained from a relationship. That is, the more satisfied in their relationship with their spouse, the more likely they are to have a healthier response to stressful situations.

An example of a potentially stressful experience is the evaluation of performance. Specific research on the effects of an unfamiliar observer on cognitive functioning suggests higher levels of performance anxiety and poorer performance on tests of attention, memory, and executive functions than performance under no observation (Horwitz & McCaffrey, 2008). Such detrimental effects on cognitive performance are present even when the observer is a parent, sibling, close friend, spouse, or partner of the individual taking the tests (Kehrer et al., 2000). Therefore, the presence of an observer may be treated as an inherent evaluator of performance, increasing subjective feelings of stress.

An index of performance monitoring that appears sensitive to others’ evaluation is the error-related negativity (ERN), an event-related brain potential (ERP) that is larger (i.e., more negative) for error response than correct responses (Falkenstein et al., 1991; Gehring et al., 1993; Gehring et al., 2012; Larson et al., 2014). Although the precise functional significance of ERN remains unclear (Clayson et al., 2021b), ERN appears to represent the activity of a performance-monitoring system that serves to adjust goal-directed behavior (Weinberg et al., 2015), which is consistent with relationships between ERN and various cognitive and motivational processes (Larson et al., 2012; Larson et al., 2014; Olvet & Hajcak, 2008; Proudfit et al., 2013; Weinberg et al., 2015). Therefore, the magnitude of the ERN can be a useful index of attention to errors (Larson & Clayson, 2011; Larson et al., 2012) and could be sensitive to the impact of perceived spousal support on performance monitoring.

Considering that the presence of strangers leads to reduced neural responses to stress in participants (Coan et al., 2006), the relationship between ERN and stress during observational paradigms is worth dissecting. Findings on the relationship between ERN and stress are mixed, with increased acute stress levels with reduced (i.e., more positive) ERN amplitude in men (Hu et al., 2019), similar ERN amplitude between acute social stress and control conditions (Rodeback et al., 2020), and larger ERN in individuals with high levels of lifetime stress exposure, particularly social-evaluative threat (Banica et al., 2022). ERN tends to be larger when participants are observed by a confederate than when completing a task alone (Hajcak et al., 2005), although the influence of the observer’s identity was not examined. In a study in which participants were observed by a romantic partner, ERN amplitude was similar under observation and when alone (Palmwood & Simons, 2021), suggesting that the identity of the observer influences whether changes in ERN are observed under observation. However, Palmwood and Simons (2021) did not compare partner observation to observation by unfamiliar people (i.e., confederates), preventing an examination of whether the observer’s identity moderates the influence of observer evaluation on performance monitoring. It is possible that heightened feelings of stress due to the presence of a confederate contribute to overactive error monitoring under evaluation, but only when the observer is a stranger.

The error positivity (Pe) is another index of performance monitoring that might be sensitive to performance evaluation. Pe is a slow tonic waveform that peaks around 300 ms and is more positive for error responses than correct responses (Falkenstein et al., 1991; O'Connell et al., 2007). Although ERN indexes early error detection, Pe reflects later conscious processing of errors and is relatively independent of ERN (Falkenstein et al., 2000; Shalgi et al., 2009). ERN in participants differentiated between harmful and nonharmful consequences of errors inflicted upon an observer, while Pe amplitude was similar across conditions (de Bruijn et al., 2020). Pe amplitude is also reduced following acute social stress manipulations, whereas ERN amplitude is not significantly affected (Rodeback et al., 2020). Taken together, error significance appears to affect early error detection, whereas stress influences later conscious processing. Therefore, ERN and Pe might show distinct relationships with social context, reflecting different stages of performance monitoring that are differentially sensitive to social and affective factors.

Gender differences are contended in research trying to understand the interaction between spousal support and cognition (Christakou et al., 2009; Crowley et al., 2009; Lighthall et al., 2012). Husbands reported that they perceive their wives as providing more quality support than they provide (Cutrona, 1996); however, a diary study observed quality of support provided did not actually differ between genders (Neff & Karney, 2005). The diary study indicated that the timing of support was different for husbands and wives. When a partner was experiencing stress, wives provided only positive support and withheld negative criticism, whereas husbands provided positive support and criticisms (Neff & Karney, 2005). It may be a matter of the mere perception of support, and the degree to which support was felt had a greater association with well-being and marital satisfaction for wives as opposed to husbands (Acitelli & Antonucci, 1994).

In relation to ERN and Pe, gender differences remain unclear, with some studies showing that men, compared to women, exhibit a large ERN (Fischer et al., 2016; Fischer et al., 2017; Larson et al., 2011a), small ERN (Themanson et al., 2011), or similar ERN (Moran et al., 2012; Weinberg et al., 2010). Results are similarly mixed for Pe, with some studies showing that men, compared to women, show large Pe amplitude (Larson et al., 2011) or no differences in amplitude (Fischer et al., 2016; Themanson et al., 2011), although studies to date on Pe are sparse. Study-to-study differences may explain the conflicting findings. Meta-analytic research indicates gender moderates the ERN-anxiety relationship, such that the relationship is stronger in women than in men with larger ERN related to higher anxiety symptoms (Moser et al., 2016). Considering gender differences in ERN and in the impact of spousal support on cognition, gender could affect the influence of spousal support on performance monitoring as indexed by ERN.

The primary aim of the present study was to determine whether spousal support mitigates the impact of stress during performance monitoring under observation. Participants completed a flanker task alone, while being observed by an other-gender confederate, and while being observed by their other-gender spouse. We predicted that participants would show larger ERN when observed than when completing a task alone. We also predicted that ERN would be smaller when completing the task in the presence of a spouse than in the presence of an unfamiliar confederate, and we examined whether gender moderated this relationship. Because research on Pe and stress is sparse, we did not have specific predictions, and analyses are considered exploratory.

**Method**

**Participants**

A total of 132 people from 66 heterosexual married couples participated in the study. Inclusion criteria were 18 to 55 years old, right-handed, and native English speakers. Exclusion criteria were a psychiatric diagnosis, self-reported alcohol or substance abuse within the past year, history of learning disability, ADHD, neurological disorder (e.g., traumatic brain injury, seizure disorder, stroke), or current use of antiepileptic medication. Participants were recruited through fliers in the local community and provided written consent for all study procedures. Seven participants were excluded due to not having any EEG data that survived artifact rejection (see Electrophysiological Recording and Data Reduction section). Therefore, final study enrollment included a total of 125 participants (65 women, 60 men) from 66 couples—some couples had partial data due to exclusion of seven participants. All participants were in their first marriage and were relatively recently married (*M* = 2.2 years^[[1]](#footnote-2)^, *SD* = 4.0 years).

**Procedures**

Each person from each couple participated in a single experimental session consisting of two main parts: 1) recording of event-related potentials (ERPs) and response times during the completion of a flanker task and 2) completion of self-report questionnaires. The computerized task was administered three times: 1) baseline (no observer), 2) confederate observer (a member of the research team matched with spouse sex), and 3) spouse observer. The baseline condition was always completed first. The order of spouse completion (husband vs. wife) and the order of the observer completion (confederate vs. spouse) were counterbalanced across couples. The spouse which was not participating in the computerized task was taken to a separate room and finished the self-report questionnaire portion of the study. Each session required approximately three hours of participation per couple and couples were provided $50 monetary compensation for their time.

**Self-Report Measures**

Self-report measures were included in the study to evaluate the effects of personal (e.g., mood) and relationship factors (e.g., marital satisfaction) on study outcomes. Most relevant to the current manuscript were the Interpersonal Reactivity Index (IRI; Davis, 1980), used to assess empathy and includes four subscales: Perspective Taking (PT), Fantasy (FS), Empathic Concern (EC), and Personal Distress (PD), and the Revised Dyadic Adjustment Scale (RDAS; Busby et al., 1995), used to assess marital adjustment and satisfaction. Descriptive statistics are provided in Table 1, and analyses with self-report measures used are provided in the supplementary material.

**Experimental Tasks**

Participants completed a modified version of the flanker task; the task is described briefly here and in full in the supplementary material. Each trial comprised a congruent or an incongruent stimulus. Participants were instructed to respond as quickly and accurately as possible. Both confederate and spouse observation conditions included the observer seated just behind and to the left of the participant. Observers were given headphones that made an audible “ding” each time the participant made an error. Only the observer heard the “ding” through the headphones.

**Electrophysiological Data Recording and Reduction**

EEG data recording and reduction is described in detail in the supplementary material and briefly outlined below. EEG was using a 129-channel hydrocel geodesic sensor net and Electrical Geodesics, Inc. (EGI; Eugene, OR) amplifier system. Continuous EEG was filtered offline with half-amplitude cutoffs at .01 and 15 Hz. Epochs were extracted from 400 ms prior to the participant’s button press to 800 ms following the button press. Ocular artifact was removed using independent components analysis, and bad channels were identified and interpolated. EEG was rereferenced to an average reference, and the period from 400 ms to 200 ms prior to participant response was used for baseline adjustment. ERN was extracted as the average activity over fronto-medial sites (6 [FCz], 129 [Cz], 7, 106; for electrode configuration, see Clayson & Larson, 2013) and quantified using a time-window mean amplitude (average activity from 0 to 100 ms). Pe was extracted as the average activity over centro-parietal sites (54, 55, 61, 62 [Pz], 78, 79) and quantified using a time-window mean amplitude (average activity from 200 to 400 ms).

**Data Analysis**

**Psychometric Internal Consistency.** Estimates of psychometric internal consistency are included (Clayson, 2024b; Clayson & Miller, 2017a, 2017b; Heindorf et al., 2025), and are described in the supplementary material.

**Multilevel Models.** Multilevel models are well suited for single-trial ERP scores (Brush et al., 2018; Clayson et al., in press; Clayson & Larson, 2019; Clayson et al., 2025; Clayson et al., 2024a; Clayson et al., 2024b; Holbrook et al., 2025; Park et al., 2024; Volpert-Esmond et al., 2018; Volpert-Esmond et al., 2021) and data nested within couples (Bartle-Haring et al., 2020). Single-trial scores were nested within participants nested within married couples and multilevel models account for the nested nature of the data. These models also account for the unbalanced nature of ERP data due to trials by partially pooling information across parameters to improve their estimation (Gelman & Hill, 2006; Gelman et al., 2012). This is beneficial here, where some participants have few trials in certain sessions (alone vs. with spouse vs. with confederate).

Location-scale multilevel models ^[[2]](#footnote-3)^ were used to simultaneously estimate mean and residual variances (Walters et al., 2018), and these models have been successfully applied in studies of ERPs (Clayson et al., 2021a; Clayson et al., 2022b; Clayson et al., 2024b; Holbrook et al., 2025; for commentary on the use of these models in studies of ERPs; see Volpert-Esmond, 2022). These models expand the multilevel structure into the scale (i.e., variance) part of the model, which allows for the simultaneous modeling of means (i.e., location) and within-person variances (i.e., scale). The multilevel location-scale model allows a multilevel structure for the residual variance, including both fixed and random effects.

Multilevel location-scale models used the same predictors on the location and scale portions. The base model for ERP data included a population intercept, a fixed effect for event (correct [reference], error), random intercepts for participants and for couples, random slopes for event within participants and within couples, and covariances between random intercepts and slopes. In the base model for RTs, there was a fixed effect for congruency (congruent [reference], incongruent) instead of a fixed effect for event. Three separate models were run that additionally included 1) a fixed effect for session (alone [reference], with confederate, with spouse), 2) a fixed effect for gender (woman [reference], man), or 3) fixed effects for session and gender and their interaction. The priors used for model fitting are described in the supplementary material.

Models were fit in *R* (R Development Core Team, 2021) using the package *brms* (Bürkner, 2017, 2018), which is a front-end wrapper for *Stan* (Stan Development Team, 2021).

Leave-one-out cross-validation via Pareto smoothed importance sampling (PSIS-LOO-CV) was used to compare model fit (Vehtari et al., 2016) using the *loo* package in *R* (Vehtari et al., 2022). All post-estimation contrasts were performed on the posterior estimates from the multilevel models. Effects were interpreted when the post-estimation contrasts excluded zero from the 95% credible intervals. Scale effects are reported in standard deviations on the log scale.

**Exploratory Analyses.** Scores from self-report measures were included as covariates in the best fitting models for each dependent variable. Scores from self-report measures were first *z*-score transformed to a mean of 0 and standard deviation of 1 prior to analysis in multilevel models. The covariates were included in the highest-order interaction and all lower-order interactions for each model. Model fits were subsequently examined against the best fitting models without covariates using PSIS-LOO-CV. Exploratory analyses are described in the supplementary material.

**Results**

Summary demographic, self-report, behavioral, and ERP data are reported in Table 1. Grand average ERP waveforms are shown in Figures 1 and 2.

**Model Selection**

We first determined the best fitting models for RTs, ERN, and Pe, and the summary statistics for these model comparisons are reported in Supplementary Table 1. The best fitting model for RTs, ERN, and Pe was a model including the three-way interaction for Congruency/Event x Session x Gender; differences in expected log predictive density ($\hat{\Delta elpd}_{loo}$) were greater than 64 for RTs, 78 for ERN, and 21 for Pe in favor of the 3-way interaction models (see Supplementary Table 1). These findings indicate that the inclusion of the three-way interaction and each lower-level interaction and main effect showed the highest predictive accuracy and generalizability to other unseen data. Therefore, the model including the three-way interaction was interpreted for each dependent variable. Findings for RTs are described in the supplementary material, as the focus of the present manuscript is on ERN on Pe.

**Error-Related Negativity**

Parameter estimates from the location-scale model predicting ERN are shown in Table and Figure 4. The unit of measurement for the 95% credible intervals for ERN and Pe is μV for the location portion of the model. Post-estimation contrasts for the scale portions of the model are reported in terms of standard deviations on the log scale.

**Location.** Error trials were larger (i.e., more negative) than correct trials (95% CrI: -3.87, -3.09). Overall, ERN was smaller (i.e., less negative) when being watched by a confederate (95% CrI: 0.90, 1.10) or a spouse (95% CrI: 0.90, 1.11) than when completing the flanker task alone. ERN was similar in amplitude when being watched by a confederate and by a spouse (95% CrI: -.11, 0.10). Women and men showed similar overall ERN amplitude (95% CrI: -.50, 0.81).

There were Event x Confederate (confederate vs. alone) and Event x Spouse (spouse vs. alone) interactions, such that ΔERN (error minus correct) was larger when being observed by a confederate or a spouse than when completing the task alone (95% CrI: -.67, -.27; 95% CrI: -.69, -.29, respectively). ΔERN was similar during the confederate and spouse conditions (95% CrI: -.18, 0.23). The Event x Gender interaction did not indicate gender differences in ΔERN (95% CrI: -1.29, 0.21). Gender differences in ΔERN were not observed when completing the task alone (95% CrI: -1.31, 0.25), with a spouse (95% CrI: -1.03, 0.54), or with a confederate (95% CrI: -1.63, -.05).

The 3-way interactions did not yield differences for the confederate vs. alone (95% CrI: -.71, 0.07) or spouse vs. alone (95% CrI: -.13, 0.68) contrasts, but there were differences observed for the confederate vs. spouse contrast (95% CrI: -1.00, -.19). Women showed differences in the Event x Confederate/Spouse contrast (95% CrI: 0.25, 0.63), but there was no such effect for men. ΔERN was larger for women during the spouse condition than during the confederate condition.

**Scale.** Correct and error trials showed similar variability in ERN amplitudes (95% CrI: -.01, 0.02). Variability was larger when completing the task with a confederate (95% CrI: 0.04, 0.07) or a spouse (95% CrI: 0.03, 0.06) than when completing the flanker task alone. Variability was similar across confederate and spouse conditions (95% CrI: -.01, 0.02). Gender differences were not observed for variability in ERN scores (95% CrI: -.06, 0.05).

There were Event x Confederate (confederate vs. alone) and Event x Spouse (spouse vs. alone) interactions. There was less variability in ΔERN during the alone condition than during the confederate condition (95% CrI: 0.03, 0.09) or the spouse condition (95% CrI: 0.03, 0.09). ΔERN variability was similar across the confederate and spouse conditions (95% CrI: -.02, 0.04). The Event x Gender interaction did not indicate gender differences in ΔERN variability (95% CrI: -.02, 0.04). Gender differences in overall ERN variability were not observed when completing the task alone (95% CrI: -.01, 0.11), with a spouse (95% CrI: -.10, 0.02), or with a confederate (95% CrI: -.08, 0.04).

The 3-way interactions did not yield differences for the confederate vs. alone (95% CrI: -.08, 0.04), spouse vs. alone (95% CrI: -.09, 0.03), or confederate vs. spouse contrasts (95% CrI: -.06, 0.06).

**Interim Summary.** Overall, ERN was smaller and ΔERN was larger when being observed than when completing the task alone, and there were no gender differences in overall ERN or ΔERN. Only women showed larger ΔERN during the spouse condition than during the confederate condition.

ERN score variability was similar across correct and error trials, and there was greater overall variability in ERN and less ΔERN variability when the participant was observed than when the participant was alone. Gender differences were not observed for the scale portion of the model.

**Error Positivity**

Parameter estimates from the location-scale model predicting Pe are shown in Table 3 and Figure 4.

**Location.** Error-trial Pe was larger (i.e., more positive) than correct-trial Pe (95% CrI: 3.68, 4.51). Pe was larger when being watched by a confederate (95% CrI: 0.47, 0.66) or a spouse (95% CrI: 0.54, 0.74) than when completing the task alone, but Pe was similar in amplitude when being watched by a confederate and by a spouse (95% CrI: -.17, 0.03). Men showed larger overall Pe than women (95% CrI: 1.13, 2.12).

There was an Event x Spouse (spouse vs. alone) interaction, such that ΔPe (error minus correct) was larger when completing the task with a spouse than when completing the task alone (95% CrI: 0.16, 0.56). Differences were not observed for the Event x Confederate (confederate vs. alone) interaction (95% CrI: -.01, 0.38) or the Event x Confederate/Spouse interaction (95% CrI: -.38, 0.02). ΔPe was larger for men than for women (95% CrI: 0.02, 1.60). Gender differences in ΔPe were observed when completing the task alone (95% CrI: 0.03, 1.66) and when completing the task with a spouse (95% CrI: 0.02, 1.68), such that men showed larger ΔPe than women during alone and spouse conditions. Gender differences were not observed for the confederate condition (95% CrI: -.10, 1.56).

The 3-way interactions did not yield differences for the confederate vs. alone (95% CrI: -.51, 0.27), spouse vs. alone (95% CrI: -.40, 0.40), or confederate vs. spouse contrasts (95% CrI: -.53, 0.28).

**Scale.** Error trials showed more Pe variability than correct trials (95% CrI: 0.06, 0.10). Variability was larger when completing the flanker task with a confederate (95% CrI: 0.02, 0.05) or a spouse (95% CrI: 0.04, 0.07) than when completing the task alone, and variability in overall Pe scores was smaller when completing the task with a spouse than with a confederate (95% CrI: -.04, -.01). Women showed more variability in Pe scores than men (95% CrI: -.13, -.004).

The Event x Spouse (spouse vs. alone) interaction indicated greater ΔPe variability when completing the task with a spouse than completing the task alone (95% CrI: 0.001, 0.06). The Event x Confederate (confederate vs. alone) and Event x Confederate/Spouse interactions did not yield differences (95% CrI: -.01, 0.04; 95% CrI: -.04, 0.01, respectively). The Event x Gender interaction did not indicate gender differences ΔPe variability (95% CrI: -.02, 0.06). Gender differences in ΔPe variability were not observed when completing the task alone (95% CrI: -.03, 0.07), with a spouse (95% CrI: -.01, 0.09), or with a confederate (95% CrI: -.05, 0.05).

The 3-way interactions did not yield differences for the confederate vs. alone (95% CrI: -.08, 0.04), spouse vs. alone (95% CrI: -.04, 0.08), or confederate vs. spouse contrasts (95% CrI: -.10, 0.02).

**Interim Summary.** Overall, Pe was larger when being observed than when completing the task alone. ΔPe was larger when completing the task with a spouse than when completing the task alone (differences were not observed for confederate vs. spouse or confederate vs. alone). Regarding gender differences, men showed larger overall Pe and ΔPe than women, gender differences in ΔPe were specific to alone and spouse conditions.

Error-trial variability was higher than correct-trial variability for Pe, and there was greater variability when completing the task with an observer than when completing the task alone. Women showed more variability in overall Pe than men. There was more variability in ΔPe when completing the task with a spouse than when completing the task alone (differences were not observed for confederate vs. spouse or confederate vs. alone).

**Discussion**

The present study examined whether the presence of a spouse attenuates neural measures of performance monitoring relative to a confederate observer and whether gender moderates these relationships. Contrary to predictions, ERN, an index of early error detection, was smaller under both spousal and confederate observation than during solitary task performance, suggesting that the presence of an observer, regardless of their relationship with the participant, reduces attention to errors. Only women showed an enhanced ERN in the presence of a spouse, an effect in the opposite direction than was predicted. Furthermore, results showed that Pe, a measure of conscious error awareness, was larger during observed conditions, regardless of the type of observer. These findings highlight the role that social context plays during performance monitoring under evaluation, offering implications for how people perceive and react to errors in the presence of others.

ERN was smaller under confederate and spousal observation than when alone, suggesting that any observer leads to reduced attention to errors. This reduction may occur because being observed introduces cognitive demands, such as concerns about social evaluation, which divert attention from error monitoring. Since high anxiety is linked to larger ERN (Hajcak et al., 2019), present findings suggest reduced neural responses to errors during social evaluation under observation by a spouse or other-gender confederate (Coan et al., 2006). Present findings contradict smaller studies, one showing larger ERN under same-gender confederate observation (*n* = 18; Hajcak et al., 2005) and another showing similar ERN amplitudes when observed by a romantic partner or when alone (*n* = 28; Palmwood & Simons, 2021). Hajcak et al. used mostly male participants and male confederates, while Palmwood and Simons lacked a confederate condition, limiting their ability to examine the impact of observer’s identity. In contrast, the present study used both an other-gender confederate and an other-gender spouse, and methodological differences (e.g., sample size, gender dynamics) may account for discrepancies with the two studies.

Notably, differences in ERN between the spouse and confederate observer conditions were only observed for women; women showed larger ΔERN in the presence of a spouse than in the presence of a confederate. This suggests that women may be more sensitive to spousal observation than men, possibly leading to greater attention to mistakes as indexed by ERN. Considering that the gender groups reported similar marital adjustment and satisfaction on the RDAS, and exploratory analyses showed that RDAS scores were not related to ERN, it seems unlikely that poor spousal support contributed to observed gender differences. Present findings are inconsistent with observations that women benefit more from spousal support than men during times of stress (Acitelli & Antonucci, 1994; Neff & Karney, 2005), indicating that the effect of spousal observation on performance monitoring likely involve gender-specific factors, such as the gender of the participant, spouse, and confederate.

Pe findings provide further context for interpreting ERN findings. ΔPe was larger when completing the task with a spouse than when alone, suggesting that participants demonstrated increased later awareness of errors when being observed by a spouse. Since the Pe reflects later, conscious error processing and the affective evaluation of errors (Overbeek et al., 2005; Shalgi et al., 2009), this increase might be due to heightened concern about a spouse’s perception. The fact that Pe did not increase under confederate observation indicates that the identity of the observer plays a crucial role in affecting conscious error processing. Gender differences were observed with men showing a larger Pe than women, and this finding is consistent with one study (Larson et al., 2011b), although this finding is inconsistent (Härpfer et al., 2020; Hsieh et al., 2021; Themanson et al., 2011). Further research on spousal support could clarify how gender moderates the relationship between spousal support and performance monitoring.

The present study had limitations. First, the “feeling” of being observed was not directly measured. Several studies have used financial (Koban et al., 2012; Koban et al., 2010; Varkala, 2023) and physically aversive (de Bruijn et al., 2020) impacts on the observer as a result of a participant’s performance to make the presence of an observer salient. Objective measures of stress were not collected, and it is plausible that some individuals might have felt positive emotions while their spouse was in the room. Second, although it is common to use a single task to record ERPs, different tasks can moderate ERP effects (Clayson, Rocha, et al., 2023; Clayson, 2024), and other tasks might show stronger (or weaker) gender differences as they relate to observers; research might consider multiverse-like approaches that consider many reasonable approaches for recording and analyzing the same phenomena (Clayson, 2024a). Third, participants were relatively young and early in their married relationships, limiting the generalizability of the findings; future research might consider additional information about marriages, such as number of years married. Fourth, the study was not preregistered, and follow-up research might consider using a registered report format (Clayson et al., 2022a). Finally, participants were excluded if they self-reported current mental illness, but lifetime psychiatric diagnoses was not assessed. Despite these limitations, the present study demonstrated several strengths, including a large sample size for an ERP study (Clayson et al., 2019; Kissel & Friedman, 2023) and the first examination of how gender moderates performance-monitoring ERP components in married participants.

Taken together, the present findings provide evidence that the presence of observers, including spouses, attenuates neural responses associated with early error detection, as evidenced by reduced ERN in observed conditions, and enhanced Pe when observed by a spouse, but not a confederate. Only women showed larger ΔERN during the spouse condition than during the confederate condition, and men showed larger ΔPe during alone and spouse conditions. Present findings also highlight the need to consider gender differences to understand the impact of an observer on performance monitoring. This study expands our understanding of social influences on performance monitoring, offering insights for future research at the intersection of social psychology and neuroscience.

**Data Availability Statement**

Data underlying this article are available from the corresponding author upon reasonable request. Because participants did not consent to public release of data, access is limited to qualified researchers for purposes such as verifying the findings, in line with APA ethical guidelines.

**Acknowledgements**

The authors have no known conflicts of interest.

References

Ainsworth, M. D. S., Blehar, M. C., Waters, E., & Wall, S. N. (2015). *Patterns of Attachment: A Psychological Study of the Strange Situation*. Psychology Press. <https://doi.org/10.4324/9780203758045>

Acitelli, L. K., & Antonucci, T. C. (1994). Gender differences in the link between marital support and satisfaction in older couples. *Journal of personality and social psychology*, *67*(4), 688.

Ainsworth, M. D. S., Blehar, M. C., Waters, E., & Wall, S. N. (2015). *Patterns of attachment: A psychological sutdy of the strange situation* (1st ed.). Psychology Press. <https://doi.org/10.4324/9780203758045>

Banica, I., Sandre, A., Shields, G. S., Slavich, G. M., & Weinberg, A. (2022). Associations between lifetime stress exposure and the error-related negativity (ERN) differ based on stressor characteristics and exposure timing in young adults. *Cognitive, Affective, & Behavioral Neuroscience*, *22*(4), 672-689. <https://doi.org/10.3758/s13415-021-00883-z>

Bartle-Haring, S., Washburn-Busk, M., & VanBergen, A. (2020). A primer for the use of multilevel models in couple and family therapy research. *Journal of Marital and Family Therapy*, *46*(4), 582-602. <https://doi.org/https://doi.org/10.1111/jmft.12433>

Brush, C. J., Ehmann, P. J., Hajcak, G., Selby, E. A., & Alderman, B. L. (2018). Using multilevel modeling to examine blunted neural responses to reward in major depression. *Biol Psychiatry Cogn Neurosci Neuroimaging*, *3*(12), 1032-1039. <https://doi.org/10.1016/j.bpsc.2018.04.003>

Bürkner, P.-C. (2017). brms: An R package for Bayesian multilevel models using Stan. *Journal of Statistical Software*, *80*, 1-28. <https://doi.org/10.18637/jss.v080.i01>

Bürkner, P.-C. (2018). Advanced Bayesian multilevel modeling with the R Package brms. *R Journal*, *10*(1), 395-411. <https://doi.org/10.32614/RJ-2018-017>

Busby, D. M., Christensen, C., Crane, D. R., & Larson, J. H. (1995). A revision of the Dyadic Adjustment Scale for use with distressed and nondistressed couples: Construct hierarchy and mutlidimensaionl scales. *Journal of Marital and Family Therapy*, *21*(3), 289-308. <https://doi.org/https://doi.org/10.1111/j.1752-0606.1995.tb00163.x>

Christakou, A., Halari, R., Smith, A. B., Ifkovits, E., Brammer, M., & Rubia, K. (2009). Sex-dependent age modulation of frontostriatal and temporo-parietal activation during cognitive control. *Neuroimage*, *48*(1), 223-236.

Clayson, P. E. (2024a). Beyond single paradigms, pipelines, and outcomes: Embracing multiverse analyses in psychophysiology. *International Journal of Psychophysiology*, *197*, 112311. <https://doi.org/10.1016/j.ijpsycho.2024.112311>

Clayson, P. E. (2024b). The psychometric upgrade psychophysiology needs. *Psychophysiology*, *61*(3), e14522. <https://doi.org/10.1111/psyp.14522>

Clayson, P. E., Baldwin, S. A., & Larson, M. J. (in press). Stability of performance monitoring with prolonged task performance: A study of error-related negativity and error positivity. *Psychophysiology*, e14731. <https://doi.org/https://doi.org/10.1111/psyp.14731>

Clayson, P. E., Brush, C. J., & Hajcak, G. (2021a). Data quality and reliability metrics for event-related potentials (ERPs): The utility of subject-level reliability. *International Journal of Psychophysiology*, *165*, 121-136. <https://doi.org/10.1016/j.ijpsycho.2021.04.004>

Clayson, P. E., Kappenman, E. S., Gehring, W. J., Miller, G. A., & Larson, M. J. (2021b). A commentary on establishing norms for error-related brain activity during the arrow flanker task among young adults. *NeuroImage*, *234*, 117932. <https://doi.org/10.1016/j.neuroimage.2021.117932>

Clayson, P. E., Keil, A., & Larson, M. J. (2022a). Open science in human electrophysiology. *International Journal of Psychophysiology*, *174*, 43-46. <https://doi.org/https://doi.org/10.1016/j.ijpsycho.2022.02.002>

Clayson, P. E., & Larson, M. J. (2013). Adaptation to emotional conflict: Evidence from a novel face emotion paradigm. *PLoS ONE*, *8*(9), e75776. <https://doi.org/10.1371/journal.pone.0075776>

Clayson, P. E., & Larson, M. J. (2019). The impact of recent and concurrent affective context on cognitive control: An ERP study of performance monitoring. *International Journal of Psychophysiology*, *143*, 44-56. <https://doi.org/10.1016/j.ijpsycho.2019.06.007>

Clayson, P. E., McDonald, J. B., Park, B., Holbrook, A., Baldwin, S. A., Riesel, A., & Larson, M. J. (2025). Registered replication report of the construct validity of the error-related negativity (ERN): A multi-site study of task-specific ERN correlations with internalizing and externalizing symptoms. *Psychophysiology*, *62*(1), e14496. <https://doi.org/10.1111/psyp.14496>

Clayson, P. E., & Miller, G. A. (2017a). ERP Reliability Analysis (ERA) Toolbox: An open-source toolbox for analyzing the reliability of event-related potentials. *International Journal of Psychophysiology*, *111*, 68-79. <https://doi.org/10.1016/j.ijpsycho.2016.10.012>

Clayson, P. E., & Miller, G. A. (2017b). Psychometric considerations in the measurement of event-related brain potentials: Guidelines for measurement and reporting. *International Journal of Psychophysiology*, *111*, 57-67. <https://doi.org/10.1016/j.ijpsycho.2016.09.005>

Clayson, P. E., Rocha, H. A., Baldwin, S. A., Rast, P., & Larson, M. J. (2022b). Understanding the error in psychopathology: Notable intraindividual differences in neural variability of performance monitoring. *Biological Psychiatry: Cognitive Neuroscience and Neuroimaging*, *7*(6), 555-565. <https://doi.org/10.1016/j.bpsc.2021.10.016>

Clayson, P. E., Rocha, H. A., McDonald, J. B., Baldwin, S. A., & Larson, M. J. (2024a). A registered report of a two-site study of variations of the flanker task: ERN experimental effects and data quality. *Psychophysiology*, *61*, e14607. <https://doi.org/10.1111/psyp.14607>

Clayson, P. E., Shuford, J., Rast, P., Baldwin, S. A., Weissman, D. H., & Larson, M. J. (2024b). Normal congruency sequence effects in psychopathology: A behavioral and electrophysiological examination using a confound-minimized design. *Psychophysiology*, *61*(1), e14426. <https://doi.org/10.1111/psyp.14426>

Coan, J. A., Schaefer, H. S., & Davidson, R. J. (2006). Lending a hand: Social regulation of the neural response to threat. *Psychological Science*, *17*(12), 1032-1039. <https://doi.org/10.1111/j.1467-9280.2006.01832.x>

Crowley, M. J., Wu, J., Crutcher, C., Bailey, C. A., Lejuez, C., & Mayes, L. C. (2009). Risk-taking and the feedback negativity response to loss among at-risk adolescents. *Developmental neuroscience*, *31*(1-2), 137-148.

Cutrona, C. E. (1996). Social support as a determinant of marital quality. In *Handbook of social support and the family* (pp. 173-194). Springer.

Davis, M. H. (1980). A multidimensional approach to individual differences in empathy. *JSAS Catalog of Selected Documents in Psychology*, *10*, 85.

de Bruijn, E. R., Jansen, M., & Overgaauw, S. (2020). Enhanced error-related brain activations for mistakes that harm others: ERP evidence from a novel social performance-monitoring paradigm. *Neuroimage*, *204*, 116238.

Falkenstein, M., Hohnsbein, J., Hoormann, J., & Blanke, L. (1991). Effects of crossmodal divided attention on late ERP components. II. Error processing in choice reaction tasks. *Electroencephalography and clinical neurophysiology*, *78*(6), 447-455.

Falkenstein, M., Hoormann, J., Christ, S., & Hohnsbein, J. (2000). ERP components on reaction errors and their functional significance: a tutorial. *Biological psychology*, *51*(2-3), 87-107.

Fischer, A. G., Danielmeier, C., Villringer, A., Klein, T. A., & Ullsperger, M. (2016). Gender influences on brain responses to errors and post-error adjustments. *Scientific reports*, *6*(1), 24435.

Fischer, A. G., Klein, T. A., & Ullsperger, M. (2017). Comparing the error‐related negativity across groups: The impact of error‐and trial‐number differences. *Psychophysiology*, *54*(7), 998-1009.

Gehring, W. J., Goss, B., Coles, M. G., Meyer, D. E., & Donchin, E. (1993). A neural system for error detection and compensation. *Psychological science*, *4*(6), 385-390.

Gehring, W. J., Liu, Y., Orr, J. M., & Carp, J. (2012). The error-related negativity (ERN/Ne).

Gelman, A., & Hill, J. (2006). *Data analysis using regression and multilevel/hierarchical models*. Cambridge University Press.

Gelman, A., Hill, J., & Yajima, M. (2012). Why we (usually) don’t have to worry about multiple comparisons. *Journal of Research on Educational Effectiveness*, *5*(2), 189-211. <https://doi.org/https://doi.org/10.1080/19345747.2011.618213>

Hajcak, G., Klawohn, J., & Meyer, A. (2019). The utility of event-related potentials in clinical psychology. *Annual Review of Clinical Psychology*, *15*, 71-95.

Hajcak, G., Moser, J. S., Yeung, N., & Simons, R. F. (2005). On the ERN and the significance of errors. *Psychophysiology*, *42*, 151-160.

Härpfer, K., Carsten, H. P., Spychalski, D., Kathmann, N., & Riesel, A. (2020). Were we erring? The impact of worry and arousal on error‐related negativity in a non‐clinical sample. *Psychophysiology*, *57*(11), e13661.

Heindorf, G., Holbrook, A., Park, B., Light, G. A., Rast, P., Foti, D., Kotov, R., & Clayson, P. E. (2025). Impact of ERP reliability cutoffs on sample characteristics and effect sizes: Performance-monitoring ERPs in psychosis and healthy controls. *Psychophysiology*, *62*(2), e14758. <https://doi.org/https://doi.org/10.1111/psyp.14758>

Holbrook, A., Park, B., Rast, P., Light, G. A., & Clayson, P. E. (2025). Intraindividual variability in event-related potentials in psychosis: A registered report. *Biological Psychiatry: Global Open Science*, *5*(1), 100396. <https://doi.org/10.1016/j.bpsgos.2024.100396>

Holt-Lunstad, J., Birmingham, W., & Jones, B. Q. (2008). Is there something unique about marriage? The relative impact of marital status, relationship quality, and network social support on ambulatory blood pressure and mental health. *Annals of behavioral medicine*, *35*(2), 239-244.

Horwitz, J. E., & McCaffrey, R. J. (2008). Effects of a third party observer and anxiety on tests of executive function. *Archives of Clinical Neuropsychology*, *23*, 409-417.

Hsieh, M.-T., Lu, H., Lin, C.-I., Sun, T.-H., Chen, Y.-R., & Cheng, C.-H. (2021). Effects of trait anxiety on error processing and post-error adjustments: an event-related potential study with stop-signal task. *Frontiers in human neuroscience*, *15*, 650838.

Hu, N., Hu, X., Xu, Z., Li, Q., Long, Q., Gu, Y., & Chen, A. (2019). Temporal dynamic modulation of acute stress on error processing in healthy males. *Psychophysiology*, *56*(9), e13398. <https://doi.org/https://doi.org/10.1111/psyp.13398>

Johnson, S. M., Moser, M. B., Beckes, L., Smith, A., Dalgleish, T., Halchuk, R., Hasselmo, K., Greenman, P. S., Merali, Z., & Coan, J. A. (2013). Soothing the threatened brain: Leveraging contact comfort with emotionally focused therapy. *PLoS ONE*, *8*(11), e79314. <https://doi.org/10.1371/journal.pone.0079314>

Kehrer, C. A., Sanchez, P. N., Habif, U. J., Rosenbaum, G. J., & Townes, B. D. (2000). Effects of a significant-other observer on neuropsychological test performance. *Clinical Neuropsychologist*, *14*, 67-71.

Koban, L., Pourtois, G., Bediou, B., & Vuilleumier, P. (2012). Effects of social context and predictive relevance on action outcome monitoring. *Cognitive, Affective, & Behavioral Neuroscience*, *12*, 460-478.

Koban, L., Pourtois, G., Vocat, R., & Vuilleumier, P. (2010). When your errors make me lose or win: event-related potentials to observed errors of cooperators and competitors. *Social Neuroscience*, *5*(4), 360-374.

Larson, M. J., & Clayson, P. E. (2011). The relationship between cognitive performance and electrophysiological indices of performance monitoring. *Cognitive, Affective, & Behavioral Neuroscience*, *11*, 159-171.

Larson, M. J., Clayson, P. E., & Baldwin, S. A. (2012). Performance monitoring following conflict: Internal adjustments in cognitive control? *Neuropsychologia*, *50*(3), 426-433. <https://doi.org/10.1016/j.neuropsychologia.2011.12.021>

Larson, M. J., Clayson, P. E., & Clawson, A. (2014). Making sense of all the conflict: A theoretical review and critique of conflict-related ERPs. *International Journal of Psychophysiology*, *93*(3), 283-297. <https://doi.org/10.1016/j.ijpsycho.2014.06.007>

Larson, M. J., South, M., & Clayson, P. E. (2011a). Sex differences in error-related performance monitoring. *Neuroreport*, *22*(1), 44-48.

Larson, M. J., South, M., & Clayson, P. E. (2011b). Sex differences in error-related performance monitoring. *NeuroReport*, *22*, 44-48. <https://doi.org/10.1097/WNR.0b013e3283427403>

Lighthall, N. R., Sakaki, M., Vasunilashorn, S., Nga, L., Somayajula, S., Chen, E. Y., Samii, N., & Mather, M. (2012). Gender differences in reward-related decision processing under stress. *Social cognitive and affective neuroscience*, *7*(4), 476-484.

Moran, T. P., Taylor, D., & Moser, J. S. (2012). Sex moderates the relationship between worry and performance monitoring brain activity in undergraduates. *International Journal of Psychophysiology*, *85*(2), 188-194.

Moser, J. S., Moran, T. P., Kneip, C., Schroder, H. S., & Larson, M. J. (2016). Sex moderates the association between symptoms of anxiety, but not obsessive compulsive disorder, and error‐monitoring brain activity: A meta‐analytic review. *Psychophysiology*, *53*(1), 21-29.

Neff, L. A., & Karney, B. R. (2005). Gender differences in social support: A question of skill or responsiveness? *Journal of personality and social psychology*, *88*(1), 79.

O'Connell, R. G., Dockree, P. M., Bellgrove, M. A., Kelly, S. P., Hester, R., Garavan, H., Robertson, I. H., & Foxe, J. J. (2007). The role of cingulate cortex in the detection of errors with and without awareness: a high‐density electrical mapping study. *European Journal of Neuroscience*, *25*(8), 2571-2579.

Olvet, D. M., & Hajcak, G. (2008). The error-related negativity (ERN) and psychopathology: Toward an endophenotype. *Clinical Psychology Review*, *28*, 1343-1354. <https://doi.org/10.1016/j.cpr.2008.07.003>

Overbeek, T. J. M., Nieuwenhuis, S., & Ridderinkhof, K. R. (2005). Dissociable components of error processing: On the functional significance of the Pe vis-à-vis the ERN/Ne. *Journal of Psychophysiology*, *19*, 319-329. <https://doi.org/10.1027/0269-8803.19.4.319>

Palmwood, E. N., & Simons, R. F. (2021). Unsupportive romantic partner behaviors increase neural reactivity to mistakes. *International Journal of Psychophysiology*, *170*, 12-19.

Park, B., Holbrook, A., Lutz, M. C., Baldwin, S. A., Larson, M. J., & Clayson, P. E. (2024). Task-specific relationships between error-related ERPs and behavior: Flanker, Stroop, and Go/Nogo tasks. *International Journal of Psychophysiology*, *204*, 112409. <https://doi.org/10.1016/j.ijpsycho.2024.112409>

Proudfit, G. H., Inzlicht, M., & Mennin, D. S. (2013). Anxiety and error monitoring: The importance of motivation and emotion. *Frontiers In Human Neuroscience*, *7*, 636. <https://doi.org/10.3389/fnhum.2013.00636>

R Development Core Team. (2021). *R: A language and environment for statistical computing*. In *R Foundation for Statistical Computing* <http://www.R-project.org/>

Rodeback, R. E., Hedges-Muncy, A., Hunt, I. J., Carbine, K. A., Steffen, P. R., & Larson, M. J. (2020). The association between experimentally induced stress, performance monitoring, and response inhibition: An event-related potential (ERP) analysis. *Frontiers In Human Neuroscience*, *14*. <https://doi.org/10.3389/fnhum.2020.00189>

Shalgi, S., Barkan, I., & Deouell, L. Y. (2009). On the positive side of error processing: Error-awareness positivity revisited. *European Journal of Neuroscience*, *29*(7), 1522-1532. <https://doi.org/10.1111/j.1460-9568.2009.06690.x>

Stan Development Team. (2021). Stan Modeling Language Users Guide and Reference Manual v2.26.1. <http://mc-stan.org/>

Themanson, J. R., Pontifex, M. B., Hillman, C. H., & McAuley, E. (2011). The relation of self-efficacy and error-related self-regulation. *Int J Psychophysiol*, *80*(1), 1-10. <https://doi.org/10.1016/j.ijpsycho.2011.01.005>

Varkala, K. (2023). *Observed Error Monitoring as an Index of Theory of Mind* University of South Florida].

Vehtari, A., Gabry, J., Magnusson, M., Yao, Y., Bürkner, P., Paananen, T., & Gelman, A. (2022). *loo: Efficient leave-one-out cross-validation and WAIC for Bayesian models*. In (Version R package version 2.5.1) <https://mc-stan.org/loo>

Vehtari, A., Gelman, A., & Gabry, J. (2016). Practical Bayesian model evaluation using leave-one-out cross-validation and WAIC. *Statistics and Computing*, *27*(5), 1413-1432. <https://doi.org/10.1007/s11222-016-9696-4>

Volpert-Esmond, H. I. (2022). Looking at change: Examining meaningful variability in psychophysiological measurements. *Biological Psychiatry: Cognitive Neuroscience and Neuroimaging*, *7*(6), 530-531. <https://doi.org/10.1016/j.bpsc.2022.02.006>

Volpert-Esmond, H. I., Merkle, E. C., Levsen, M. P., Ito, T. A., & Bartholow, B. D. (2018). Using trial-level data and multilevel modeling to investigate within-task change in event-related potentials. *Psychophysiology*, *55*(5), e13044. <https://doi.org/10.1111/psyp.13044>

Volpert-Esmond, H. I., Page-Gould, E., & Bartholow, B. D. (2021). Using multilevel models for the analysis of event-related potentials. *International Journal of Psychophysiology*, *162*, 145-156. <https://doi.org/10.1016/j.ijpsycho.2021.02.006>

Walters, R. W., Hoffman, L., & Templin, J. (2018). The power to detect and predict individual differences in intra-individual variability using the mixed-effects location-scale model. *Multivariate Behavioral Research*, *53*(3), 1-15. <https://doi.org/10.1080/00273171.2018.1449628>

Weinberg, A., Dieterich, R., & Riesel, A. (2015). Error-related brain activity in the age of RDoC: A review of the literature. *International Journal of Psychophysiology*, *98*(Part 2), 276-299. <https://doi.org/10.1016/j.ijpsycho.2015.02.029>

Weinberg, A., Olvet, D. M., & Hajcak, G. (2010). Increased error-related brain activity in generalized anxiety disorder. *Biol Psychol*, *85*(3), 472-480. <https://doi.org/10.1016/j.biopsycho.2010.09.011>

Figure Captions

Figure 1. Grand average waveforms for the error-related negativity (ERN) separately for men (top row) and women (bottom row). Waveforms are shown for fronto-central activity across four sites for ERN on the top row, and activity corresponding to ERN is shown in gray. Topographical maps showing the frontocentral scalp distribution of ERN for each group are shown in the top-right corner of each plot, and the maps represent the average ERN difference activity (error minus correct) from 0 to 100 ms for waveforms during the alone condition.

Figure 2. Grand average waveforms for the error positivity (Pe) separately for men (top row) and women (bottom row). Waveforms are shown for centro-parietal activity across six sites for Pe, and activity corresponding to Pe is shown in gray. Topographical maps showing the frontocentral scalp distribution of Pe for each group are shown in the top-right corner of each plot, and the maps represent the average Pe difference activity (error minus correct) from 200 to 400 ms for waveforms during the alone condition.

Figure 3. Point estimates for posterior samples with their 95% credible intervals. Estimates of average error-related negativity (ERN; top left column) and error positivity (Pe; top right column) are shown on the top, and estimates of the ERN standard deviations (bottom left) and Pe standard deviations (bottom right) on the log scale are shown on the bottom.

1. Data were missing for one couple. [↑](#footnote-ref-2)
2. A discussion of location-scale models and their application to ERPs is described in the supplementary material of Clayson et al. (2022). [↑](#footnote-ref-3)
